# Changes in Emotional Regulation, Well-Being, and Pain Perception Following Music Therapy in People with Sensory Processing Sensitivity: A Non-Controlled Exploratory Study

**DOI:** 10.3390/healthcare14162509

**Published:** 2026-08-12

**Authors:** Alicia Francisca Ponce-Valencia, Diana Jiménez Rodríguez, Leonor Alberola Amores, Alba Álvarez Pujante, Manuela Pérez-Chacón, Antonio Chacón, Paloma Echevarría Pérez

**Affiliations:** 1 Facultad de Enfermería, Universidad Católica de Murcia, Campus de Guadalupe, Avda, de Los Jerónimos, 135, 30107 Murcia, Spain; aponce@ucam.edu (A.F.P.-V.); albapujante_0401@icloud.com (A.Á.P.); pechevarria@ucam.edu (P.E.P.); 2 Facultad Ciencias de la Salud, Universidad de Almería, Carretera Sacramento, s/n, La Cañada de San Urbano, 04120 Almería, Spain; d.jimenez@ual.es; 3 Spanish Association of Highly Sensitive Professionals and Psychologists, PAS España, 11500 El Puerto de Santa María, Spain; manuelaperezchacon@gmail.com (M.P.-C.); antonio.chacon@pasespana.org (A.C.)

**Keywords:** highly sensitive people, music therapy, sound therapy, gong, care, well-being, pain

## Abstract

**Background/Objectives:** Highly sensitive persons (HSPs) are characterized by increased emotional and sensory responsiveness, which may predispose them to anxiety, emotional dysregulation, and sensory overload. Music therapy based on gong sound sessions has emerged as a complementary intervention aimed at promoting emotional well-being. This study analyzed immediate pre- and post-intervention variations in an adult population with sensory processing sensitivity. **Methods:** A quantitative, quasi-experimental, single-group (non-controlled), pre–post intervention study was conducted using music therapy in a sample of N = 93 individuals, who were evaluated during an initial session (62 Highly Sensitive Persons [HSPs] and 31 Non-HSPs), from which a synchronized longitudinal sub-cohort of 39 participants (27 HSPs and 12 Non-HSPs) completed a second exposure. Indicators were assessed using the Questionnaire on the Impact of Music Therapy Sessions in Adult Patients (CISMA), whose technical designation corresponds to an acronym of Spanish-language origin. **Results:** Participants demonstrated significant score variations following gong sound therapy in each session. In the first session, the mean CISMA score changed by 7.0 points (*p* < 0.001), while the second session showed a mean variation of 7.0 points (*p* < 0.001). Participants reported favorable trends in emotional self-regulation, reduced anxiety, mitigated pain intensity, increased tolerance to sensory overload, and greater overall psychological well-being. **Conclusions:** Music therapy using gong sound appears to be a promising complementary framework, contributing to immediate positive shifts in emotional regulation, anxiety, pain, and sensory distress. Within the limitations of this non-controlled framework, these immediate shifts describe a specific statistical variation within the observed sample, which cannot be generalized as clinical efficacy. Controlled trials are needed to confirm these insights and show the potential integration of music therapy using gong sound into holistic nursing and mental health care models.

## 1. Introduction

Sensory Processing Sensitivity (SPS) is a non-pathological personality trait characterized by enhanced sensitivity to internal and external stimuli, deeper cognitive processing, heightened emotional reactivity, and increased awareness of environmental cues [1]. Its estimated prevalence is around 15–30% in the general population [1,2].

People with this trait tend to perceive the world more intensely than most individuals, meaning that stimuli considered trivial by others may become overwhelming for them [1]. Previous works suggest that these individuals exhibit distinct patterns of brain activity associated with deeper emotional processing. Individuals with high levels of SPS, commonly referred to as Highly Sensitive People (HSPs), are characterized by strong emotional and empathic responsiveness, deeper cognitive processing, and increased awareness of environmental and social cues [3]. This heightened sensitivity may lead to overstimulation and elevated stress levels, particularly in demanding environments. Although high sensitivity was initially viewed primarily as a vulnerability [4], more recent studies indicate that individuals with high SPS may also display adaptive qualities, experiencing greater positive emotional responses in supportive environments [5]. In this sense, highly sensitive individuals show an increased responsiveness to both positive and negative experiences [6].

This particular sensitivity to the environment can have important implications in several contexts, like health, education, and others. SPS is also considered a biologically determined temperament trait associated with heightened awareness and responsiveness to environmental and social stimuli [7,8]. HSPs are often recognized as observant, empathetic, and highly creative individuals; however, they are also more vulnerable to stress, emotional exhaustion, and various mental health challenges [9]. Consequently, educational and healthcare environments can strongly influence their personal and professional development.

Although Sensory Processing Sensitivity (SPS) has received increasing attention in personality and health research, its construct validity and discriminant validity remain the subject of ongoing scientific debate [2]. Current psychometric evidence suggests that SPS should be interpreted within a critical and balanced framework, particularly regarding its distinction from established personality traits.

The main lines in personality psychology are structured around three fundamental axes:

- Overlap with Neuroticism: Various authors question whether SPS possesses sufficient distinctiveness or if, conversely, it shares excessive variance with the neuroticism (negative emotional stability) dimension of the Big Five model. Critics point out that susceptibility to distress from overstimulation could be a secondary manifestation of the stress vulnerability inherent in neuroticism, given that the subscales of the standard measurement instrument (HSP) show moderate-to-high correlations with this trait [10].
- Convergence with Introversion: The degree of overlap between SPS and classic introversion is a subject of active debate [1]. Although it is theoretically posited that SPS involves deep information processing rather than merely social withdrawal, empirically differentiating the two in self-report scales remains a complex methodological challenge, as both constructs conceptually share a tendency to avoid highly stimulating environments [11].
- Discriminant Validity: Current scrutiny focuses on determining whether available assessment tools can clearly isolate SPS from other personality traits or from clinical profiles associated with affective dysregulation and generalized stress vulnerability [6].

In highly demanding settings such as nursing, HSPs may be especially susceptible to overstimulation and emotional burnout. In this context, the integration of complementary therapies may offer significant benefits for their well-being. These approaches can contribute to stress reduction, improved emotional regulation, and the creation of healthier and more sustainable care environments [9]. Complementary or natural therapies encompass a wide range of therapeutic approaches aimed at enhancing health through natural methods. Within clinical and health practice, these modalities have been referred to by terms such as traditional health care, natural health care, alternative therapies, or complementary therapies, although all share a common focus on health promotion, disease prevention, and the strengthening of personal resources [12].

Among these approaches, music therapy has gained recognition as an effective complementary therapy. Rather than replacing conventional medical or psychological treatments, music therapy integrates with them to enhance overall therapeutic outcomes. The primary objective of this therapy is to promote emotional, physical, and mental well-being, alleviate symptoms associated with anxiety, depression, and stress, and facilitate emotional self-regulation and personal growth. Music therapy has been applied in diverse clinical and educational settings and has shown particularly beneficial effects [13].

Gong-based sound interventions have been used for centuries in different cultural traditions and have recently attracted increasing interest as complementary therapeutic approaches [11]. Preliminary evidence suggests that these interventions may facilitate relaxation, emotional regulation, and subjective well-being in some populations. However, the mechanisms through which these effects may occur remain incompletely understood and continue to be investigated [14]. Within this context, gong-based music therapy may represent a complementary intervention worthy of further study in individuals with high sensory processing sensitivity [15].

Music operates on physical, intellectual, and affective–emotional levels simultaneously, facilitating direct and immediate access to multiple dimensions of human functioning [16,17]. Through listening to music, individuals may express and release emotions, regulate mood, and promote psychological and emotional development [18]. The scientific literature indicates that music has the capacity to induce both sedative and stimulating effects [19], establishing itself as an effective therapeutic resource in the clinical management of anxiety, depression, and somatization disorders [20]. Furthermore, within theoretical frameworks, sound stimulation is described in the literature as playing a potential role in modulating subjective perceptions of brain rhythms, circulation, respiration, digestion, metabolism, muscle tone, neuronal activity, and the immune response [21].

Considering all the above, the aim of this pilot study was to examine immediate changes in self-reported emotional and sensory outcomes following gong-based music therapy in adults with and without high SPS. Immediate pre–post changes were evaluated after two intervention sessions using the Questionnaire on the Impact of Music Therapy Sessions in Adult Patients (CISMA). In addition, we sought to analyze changes in the subjective perception of pain within both HSP and non-HSP individuals, mapping the magnitude of these variations before and after each session within this exploratory framework.

## 2. Materials and Methods

### 2.1. Design

This study employed a quantitative, comparative, and longitudinal uncontrolled quasi-experimental design with a single-group pre–post framework. The research was conducted in institutional collaboration with the Association of Psychologists of High Sensitivity Professionals (PAS Spain), which is registered nationally under number 614062, located in the cities of Jerez de la Frontera, León, A Coruña, and Murcia, Spain. Given the exploratory pilot nature of this study, prospective clinical trial registry was not undertaken. The study aimed to explore the response patterns following gong-based sound therapy sessions within Highly Sensitive People (HSPs) and non-HSP individuals through repeated measures over time.

A longitudinal approach was adopted to monitor variations in participants’ perceptions before and after the music therapy framework across two sessions separated by an interval of one and a half months. The study was based on the repeated administration of the CISMA questionnaire, allowing for a detailed assessment of the evolution of emotional perception, auditory sensitivity, and anxiety reduction associated with these music therapy sessions.

The intervention consisted of two gong sound therapy sessions conducted in controlled and relaxing environments. Pre- and post-intervention assessments were administered during both sessions in order to compare participants’ responses and analyze score variations over time.

Given the absence of a non-intervention control group, this study is explicitly framed as an exploratory pilot investigation. Consequently, the observed changes pre- and post-intervention should be interpreted with caution, as confounding factors such as placebo effects, participant expectations, natural relaxation, or testing effects cannot be fully isolated.

### 2.2. Participants

The sample consisted of 130 adults divided into two groups: 90 participants identified as Highly Sensitive People (HSPs) and 40 participants without the high-sensitivity trait (non-HSP). Participants were classified using the 27-item Highly Sensitive Person Scale—Spanish version (HSPS-S) [12], corresponding to the cross-cultural adaptation of the original Highly Sensitive Person Scale developed by Aron and Aron [1] and validated for the Spanish population by Chacón et al. [22] rather than shortened versions. In accordance with the psychometric criteria established for the Spanish population, high sensory processing sensitivity was defined using the 67th percentile as the classification threshold. Participants with a total score of ≥160 for men or ≥167 for women were classified as Highly Sensitive Persons (HSPs), whereas those scoring below these thresholds were classified as non-HSPs. Data collection was carried out between December 2024 and March 2025 and non-randomised.

The inclusion criteria were: (a) being over 18 years of age, (b) being identified either as HSP or non-HSP according to the HSP scale, and (c) participating in both scheduled gong sound therapy sessions. Exclusion criteria included: (a) participants under 18 years of age and (b) individuals who did not correctly complete the CISMA questionnaire [23].

Participants were informed about the objectives and procedures of the study, and confidentiality and anonymity were guaranteed in accordance with Organic Law 3/2018 of 5 December on the Protection of Personal Data and Guarantee of Digital Rights. Participation was voluntary, without financial compensation, and completion of the questionnaire was considered as informed consent.

Complete pre- and post-intervention evaluations for the first session were obtained for 93 participants (comprising 62 HSP and 31 non-HSP individuals), after strictly excluding cases with missing post-test responses. Due to participant attrition, non-attendance, and follow-up availability across the multi-month study framework, the longitudinal tracking for the second session was conducted with a final sub-sample of 39 participants (consisting of 27 HSP and 12 non-HSP individuals) who completed all time points. No data imputation techniques were applied to missing data.

### 2.3. Therapeutic Intervention Design

The intervention consisted of two gong sound therapy sessions lasting 45 min each, separated by an interval of approximately one and a half months. An additional ten minutes were allocated before and after each session for completion of the questionnaires. Sessions for HSP participants were conducted within the facilities of their respective HSP associations, as these institutional spaces were strictly reserved for activities involving registered members. Consequently, to facilitate logistics for non-associated individuals, sessions for non-HSP participants took place in a multipurpose room at a training academy.

To minimize differences in the immediate physical environment, both locations were prepared using comparable floor mats, ambient lighting, room temperature, and noise control. Nevertheless, the social and institutional context differed between locations and may have influenced participants’ responses, representing a potential source of residual confounding.

The sound intervention employed a 32-inch Real Gong planetary (Lorquí, Spain) gong tuned to the musical note E3, corresponding to a frequency of 161.26 Hz, following Hans Cousto’s planetary tuning system based on the theoretical principles of Hans Kayser. The gong was played using M4 and M5 mallets. Additionally, 7.5-inch zill cymbals (Meinl Percussion, Gutenstetten, Germany) tuned to 4096 Hz were used at the beginning and end of the sessions to open and close the sound space. The gong performance was structured into three consecutive blocks of 15 min each, with predetermined rhythmic and vibrational patterns, including integration pauses between sequences. The intervention was designed to promote relaxation and emotional regulation through sustained harmonic sound stimulation [17].

### 2.4. Primary and Secondary Outcome

The primary outcome of the study was to evaluate the perceived impact of gong sound therapy on participants’ overall well-being, operationalized and quantified through changes in the global score of the Questionnaire on the Impact of Music Therapy Sessions in Adult Patients (CISMA) before and after the sessions. For the purposes of this investigation, the global CISMA score was calculated using a standardized scoring procedure that sums the individual categorical indices into a continuous cumulative metric ranging from a minimum of 4 to a maximum of 40 points, wherein the subjective pain metric is inversely integrated (subtracted) to ensure that higher total scores reflect a more favorable self-reported well-being state.

The secondary outcomes focused on specific dimensions in relation to pain, HSP differences with Non-HSPs, and sex variations. Changes in participants’ perceptions before and after the intervention were assessed using the individual dimensions of the CISMA questionnaire, analyzed separately according to their specific sub-scale boundaries.

### 2.5. Data Collection Instruments

Data were collected using the CISMA questionnaire (Questionnaire on the Impact of Music Therapy Sessions in Adult Patients) whose technical designation corresponds to an acronym of Spanish-language origin, a self-administered instrument designed to explore the perceived experiences and variations associated with music therapy interventions [23]. The CISMA questionnaire utilizes a consistent metric system across its sections: a 1–10 Numeric Analogue Scale (NAS) dedicated exclusively to evaluating subjective pain perception within the physical dimension, and a parallel Likert-type scale for the emotional and social functioning dimensions. The questionnaire comprises five domains: general well-being, relaxation, mood, pain intensity, and perceived companionship/social support. Each domain is scored on a 10-point scale ranging from 1 to 10, with higher scores indicating a more favourable perceived status for the construct being assessed.

For the purposes of the present study, an overall global CISMA composite score was calculated by integrating the five items. To resolve the opposite valence of these metrics into a unified index, the scoring protocol treats pain as an inverse indicator of well-being; thus, the raw score of the pain item is arithmetically subtracted from the cumulative sum of the four positive emotional and social items. Consequently, this balanced scoring procedure yields a continuous global well-being index ranging from a minimum of 4 to a maximum of 40 points, where higher total scores reflect a more favourable overall self-reported response following the intervention. In addition to the global score, each individual CISMA domain was analysed separately.

By sharing this uniform decimal baseline, the instrument easily aggregates the scores into three main dimensions of well-being, physical, emotional, and social functioning, allowing participants to provide a rapid evaluation of their general state. The physical dimension specifically incorporates the pain metric alongside items dedicated to evaluating somatic manifestations such as muscle tension and stress-related discomfort. For the present study, this global composite framework demonstrated high reliability, yielding a Cronbach’s alpha of α = 0.686 and a McDonald’s omega of ω = 0.669, across the active sample, establishing strong baseline internal consistency.

Questionnaires were administered digitally through the Google Forms platform. Participants accessed the forms using a QR code and completed the questionnaires on their own electronic devices. A researcher was present during data collection to provide instructions and resolve any doubts related to questionnaire completion. Both pre-test and post-test assessments were administered in each of the two sessions.

### 2.6. Data Analysis

Descriptive statistical analyses were conducted using frequencies and percentages for categorical variables and means and standard deviations (±SD) for continuous variables. Normality assumptions and sampling distributions were evaluated through the Kolmogorov–Smirnov test alongside visual inspection of Q-Q plots.

To address the primary longitudinal and multi-factorial objectives within this repeated-measures framework, linear mixed-effects models (LMMs) were implemented. This advanced approach was selected to effectively account for the nested structure of the data (repeated measures within subjects) and to handle the unbalanced sub-cohort sizes across sessions while retaining all available data points. However, it is acknowledged that the reduction in sub-cohort sizes across consecutive sessions inherently reduces statistical power for secondary subgroup contrasts. The analytical models specified Highly Sensitive Person (HSP) status (HSP vs. non-HSP), Time (Pre- vs. Post-intervention), and Session (Session 1 vs. Session 2) as fixed effects, including their two-way and three-way interaction terms. Subject identity was introduced as a random intercept to control for baseline inter-individual variability. Type III Analysis of Variance (ANOVA) tables were generated using Satterthwaite’s approximation for degrees of freedom. To maintain rigorous error control across multiple comparisons, *p*-values derived from post-hoc pairwise contrasts were adjusted using the False Discovery Rate (FDR) method.

To complement the global mixed-effects framework and characterize specific short-term variations within isolated sub-cohorts, paired-samples *t*-tests were utilized for direct pre–post intra-group comparisons, while independent-samples *t*-tests were applied for cross-sectional sex and cohort comparisons. The magnitudes of these standardized mean differences were quantified using Cohen’s d (interpreted as small ≥ 0.2, medium ≥ 0.5, and large ≥ 0.8) along with their corresponding 95% confidence intervals (CIs).

Statistical modeling and advanced mixed-effects frameworks were executed using R (version 4.3.1) via the lme4 and lmerTest packages, while standard descriptive data management was supported by IBM SPSS Statistics (version 27.0). For all inferential tests, statistical significance was strictly established at the conventional *p* < 0.05 threshold.

Finally, to evaluate the statistical adequacy and sensitivity of the sample sizes, a post-hoc power analysis was conducted using G*Power software (version 3.1.9.7). For the primary pre–post longitudinal contrasts (paired-samples *t*-tests), the analysis yielded a statistical power (1 − β) of approximately 1.0, confirming exceptional sensitivity to detect the observed large effect sizes. For the secondary cross-sectional comparisons between sensitivity profiles (independent-samples *t*-tests), the statistical power stood at approximately 0.70, reflecting a moderate yet acceptable capacity to detect medium-to-large differences within this exploratory framework.

## 3. Results

### 3.1. Participant Characteristics and Baseline Data

The study sample initially consisted of 130 adult participants, divided into two groups according to their level of Sensory Processing Sensitivity (SPS) using Elaine Aron’s Highly Sensitive Person Scale (HSP). Regarding the final sample flow utilized for the analyses, after excluding participants with incomplete pre–post assessments during the first intervention session, the first session yielded a total of 93 valid evaluations, which were distributed into 62 HSP and 31 non-HSP participants. The overall sample was aged between 18 and 67 years, with a mean age of 44.97 years (SD = 11.21).

**Figure 1:**
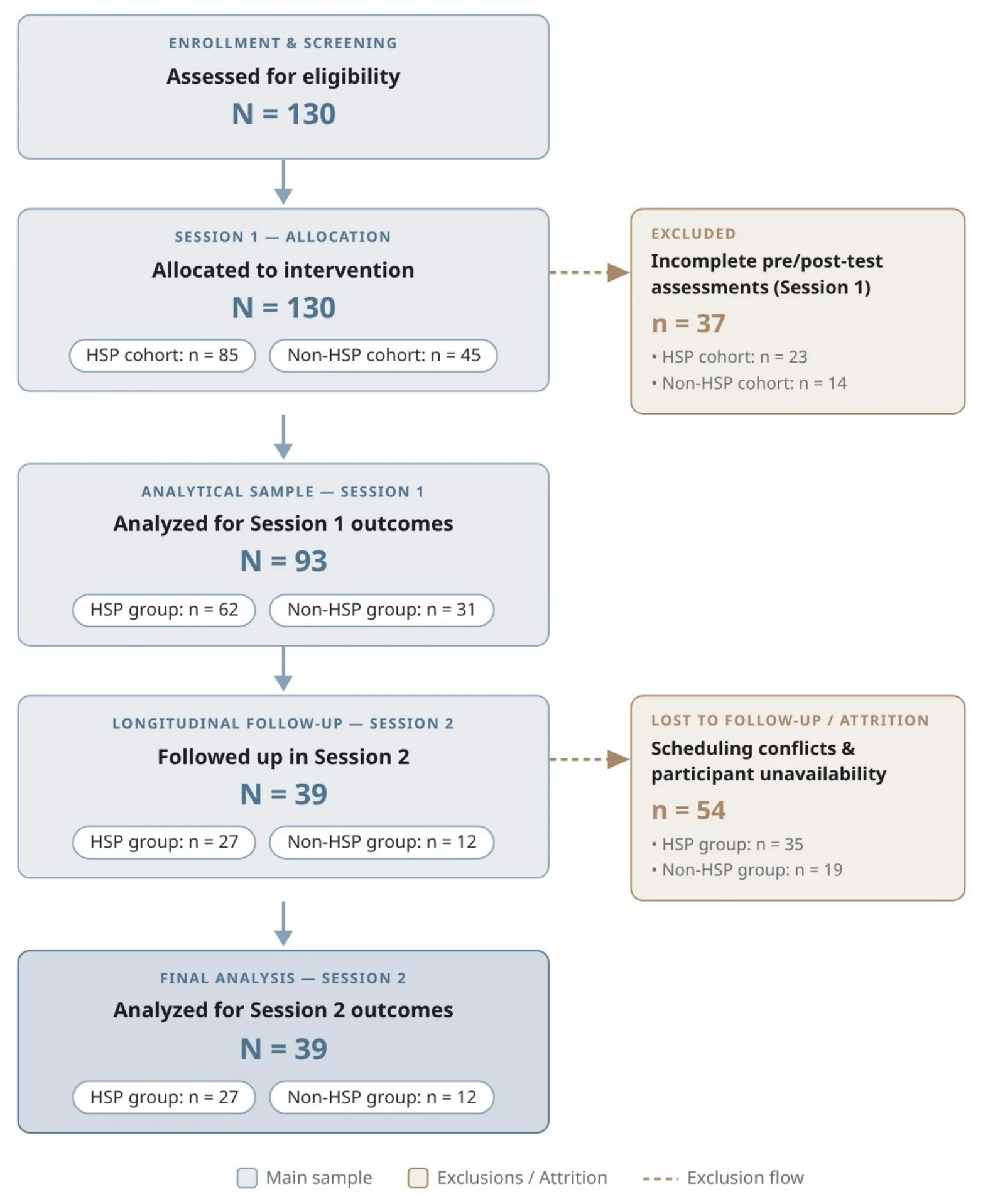
TREND-style participant flow diagram throughout the evaluation framework.

Due to longitudinal attrition across the multi-month study framework, the second session was completed by a final tracked cohort of 39 participants, comprising 27 individuals from the HSP group and 12 from the non-HSP group. No data imputation techniques were applied, ensuring that all statistical inferences reflect complete cases. The complete screening, exclusion pathways, and longitudinal tracking of the sample are visually mapped in Figure 1.

Detailed baseline demographic characteristics and initial psychometric outcomes assessed via the CISMA questionnaire for the active sample (N = 93) are structured and summarized in Table 1.

### 3.2. Pre–Post and Cross-Session Assessment Matrix

**Figure 2:**
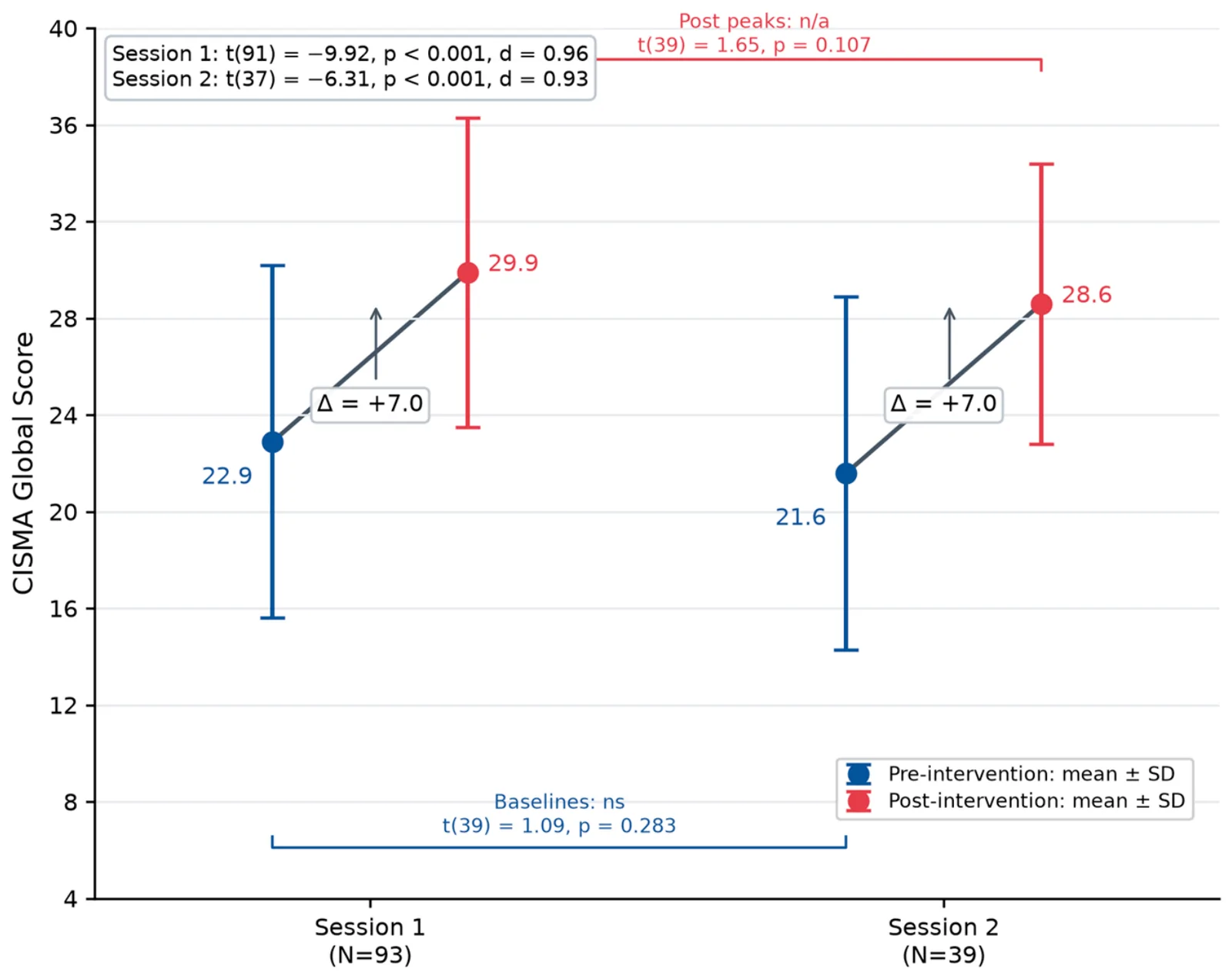
CISMA: Pre–Post and Cross-Session Assessment Matrix. The figure illustrates the study assessment protocol, including pre- and post-intervention measurements for each session and the comparison between Sessions 1 and 2. Data are presented as mean ± standard deviation (SD). Arrows indicate the magnitude and direction of the pre–post change (Δ = +7.0).

Longitudinal changes in CISMA scores were first examined using a linear mixed-effects model. The Type III Analysis of Variance (ANOVA) revealed a significant main effect for the factor Time (F (1, 160.24) = 92.11, *p* < 0.001). Within the descriptive limits of this single-arm design, this statistical observation reflects a generalized upward shift in global CISMA scores immediately following the acoustic sessions across the evaluated participants (Figure 2).

In the first session (*N* = 93), participants showed an upward shift in post-intervention global scores compared to their baseline measurements. The mean score changed from a pre-intervention baseline of 22.9 points (±7.2) on a 4–40 scale to a post-intervention score of 29.9 (±6.5), yielding an average unadjusted improvement of 7.0 points (95% CI [5.60, 8.40]). Statistical testing through a paired-samples *t*-test confirmed this pre–post difference (t(92) = 9.92, *p* < 0.001), representing a large standardized mean difference (Cohen’s *d* = 0.96). While the absence of a control group precludes definitive causal or clinical efficacy attributions, this metric reflects a substantial statistical variation in participants’ reported well-being immediately following the session.

Similarly, in the second session (*N* = 39), participants exhibited an increase in total CISMA scores following the intervention. The mean score increased from 21.6 (±7.5) to a post-intervention score of 28.6 (±6.0), representing a descriptive change of 7.0 points (95% CI [4.75, 9.25]; t(38) = 6.31, *p* < 0.001; Cohen’s d = 0.93). This indicates a consistent descriptive pattern, showing that the magnitude of the pre–post mathematical change remained stable during the second exposure.

Cross-session comparisons evaluated within the synchronized longitudinal cohort (N = 39) using paired-samples *t*-tests did not reveal statistically significant differences between baseline measurements of the first and second sessions (22.9 vs. 21.6 points, respectively; t(38) = 1.09, *p* = 0.283). Likewise, no significant differences were observed when comparing the post-intervention peaks across both exposures (29.9 vs. 28.6 points, respectively; t(38) = 1.65, *p* = 0.107). A similar pattern of statistically significant pre–post improvement was observed during the second intervention session.

### 3.3. Changes in CISMA Score in People with High Sensory Processing (HSP) or Not (No HSP)

**Figure 3:**
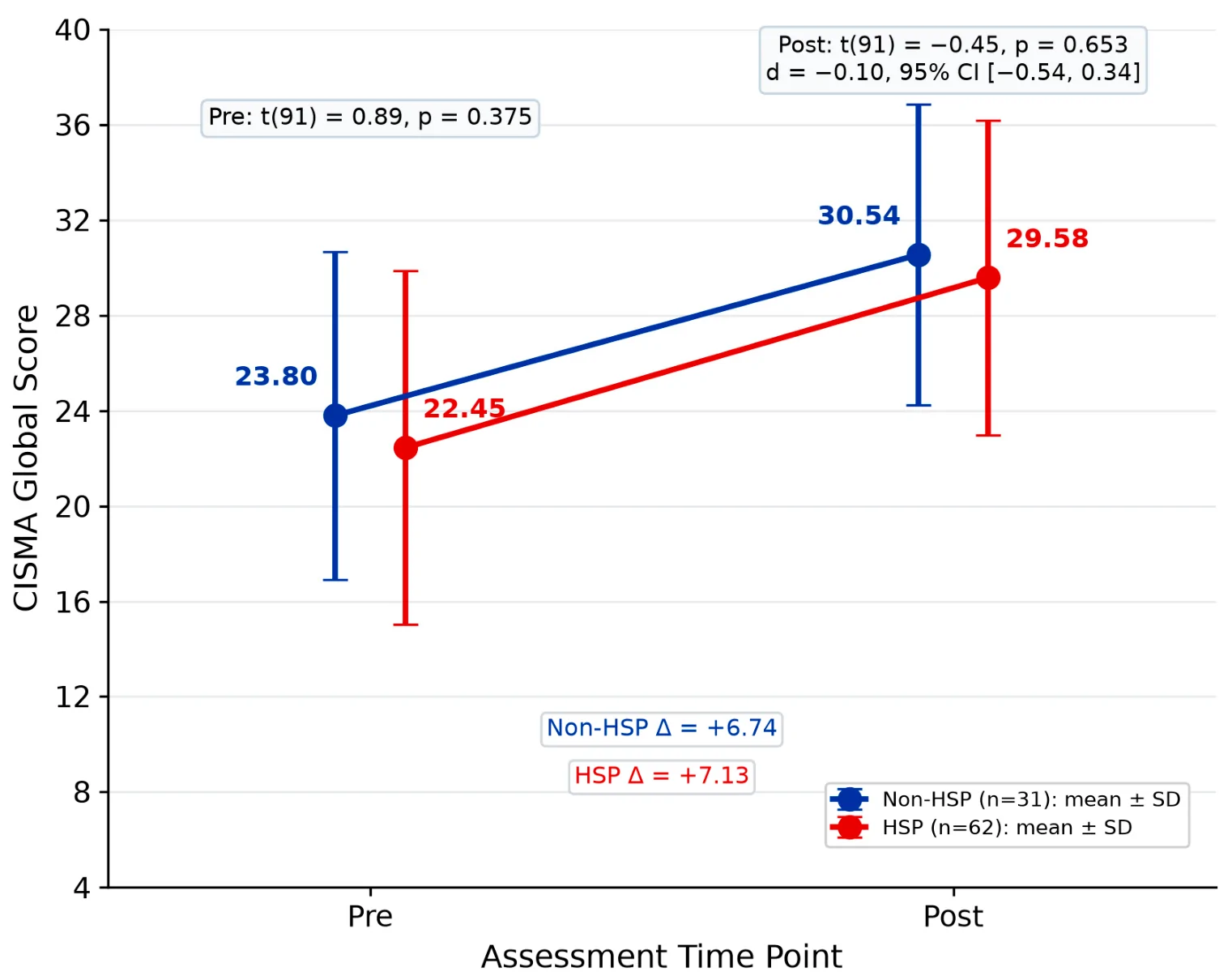
Evolution of global CISMA scores during the first intervention session. The line chart displays the mean scores (±SD) from pre- to post-intervention for Highly Sensitive Persons (HSPs, red line) and Non-Highly Sensitive Persons (non-HSPs, blue line). Inferential statistics derived from independent-samples *t*-tests are detailed for baseline (Pre) and post-test (Post) cross-sectional comparisons.

**Figure 4:**
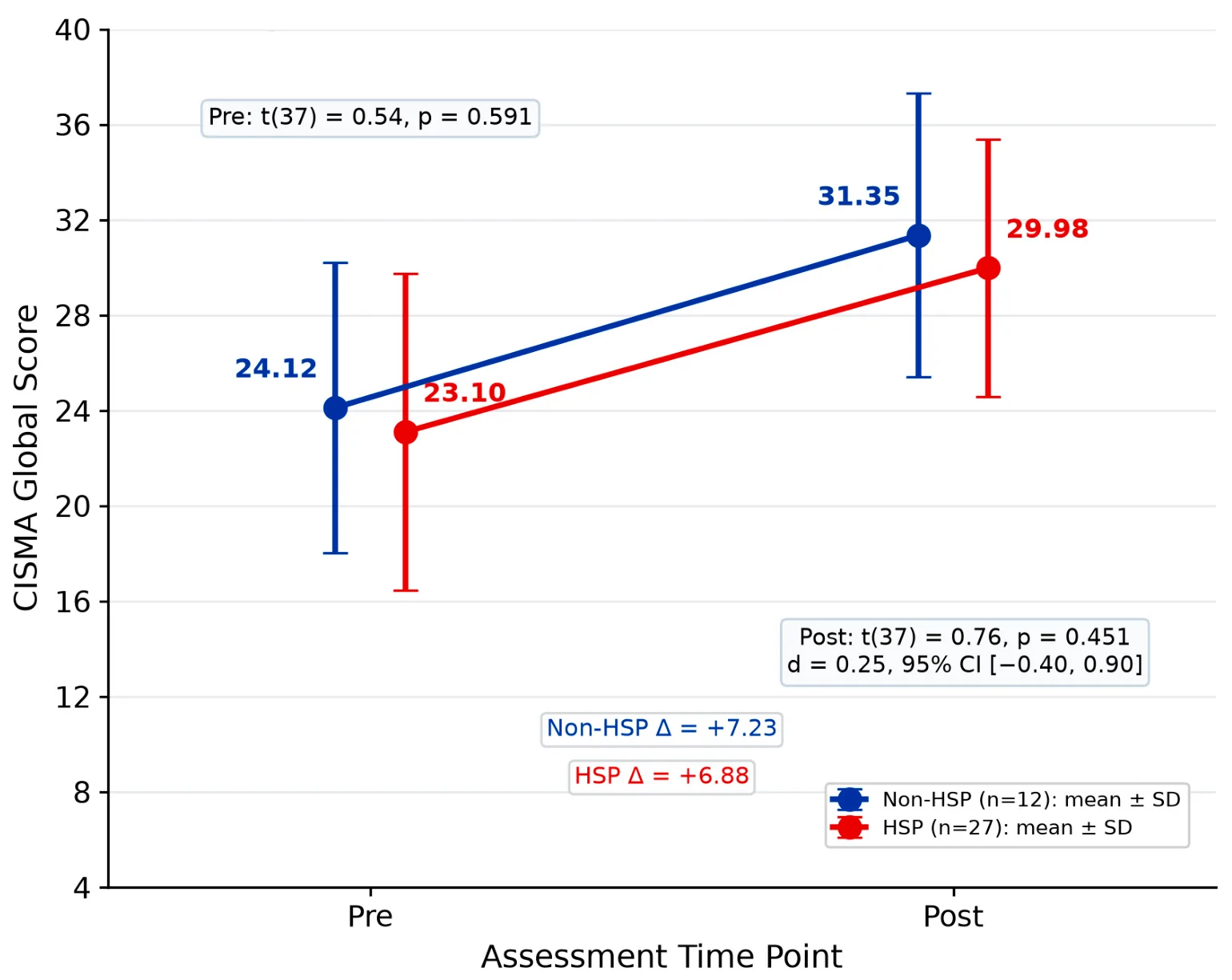
Longitudinal evolution of global CISMA scores during the second intervention session. The line chart displays the tracking of mean scores (±SD) from pre- to post-intervention for both sub-cohorts within the longitudinal sample framework. Cross-sectional group comparisons and standardized effect sizes (Cohen’s d with 95% confidence intervals) are specified at each assessment time point.

Regarding the processing sensitivity traits, the global linear mixed-effects model indicated no significant main effect for HSP status (*p* = 0.585) and no significant Time x HSP interaction (*p* = 0.351). These metrics indicate that the observed upward shifts developed along fundamentally parallel trajectories in both sub-cohorts. To further characterize the cross-sectional data distributions at specific points, exploratory independent-samples *t*-tests were conducted by segregating participants into Non-Highly Sensitive Persons (Non-HSP) and Highly Sensitive Persons (HSP) (Figure 3 and Figure 4). As detailed in the study limitations, these statistical comparisons are purely descriptive due to the logistical confounding between group membership and physical venues.

During the baseline measurement (Pre) of the first session, no statistically significant differences were observed between Non-HSP (n = 31, M = 23.80 ± 6.88) and HSP (n = 62, M = 22.45 ± 7.42; t(91) = 0.89, *p* = 0.375), indicating homogenous initial levels between sub-cohorts. Immediately following the sound experience (Post), both groups exhibited descriptive upward shifts, with Non-HSP reaching a mean score of 30.54 (±6.30) and HSP achieving 29.58 (±6.60). An independent-samples *t*-test detailed that post-intervention scores did not differ significantly between sensitivity profiles (t(91) = −0.45, *p* = 0.653), displaying a negligible standardized mean difference (Cohen’s d = −0.10, 95% CI [−0.54, 0.34]). This pattern suggests a shared descriptive variation trend regardless of processing sensitivity.

In the second session, evaluated within the longitudinal sub-cohort, baseline scores remained statistically comparable between Non-HSP (n = 12, M =24.12 ± 6.10) and HSP (n = 27, M = 23.10 ± 6.65; t(37) = 0.54, *p* = 0.591). Following the second exposure, post-intervention metrics rose to 31.35 (±5.95) in the Non-HSP group and 29.98 (±5.40) in the HSP group. The independent-samples *t*-test indicated that no statistically significant differences were observed between the groups after the second intervention session (t(37) = 0.76, *p* = 0.451), displaying a small standardized mean difference (Cohen’s d = 0.25, 95% CI [−0.40, 0.90]).

In summary, within the descriptive limits of this non-controlled quasi-experimental framework and its inherent contextual confounding, changes in immediate well-being scores developed progressively and uniformly across both sensitivity profiles, without showing statistical indication of an interaction or distinct filtering attributable to the HSP trait.

### 3.4. Changes in CISMA Score Depending on Sex of Study Participants

**Figure 5:**
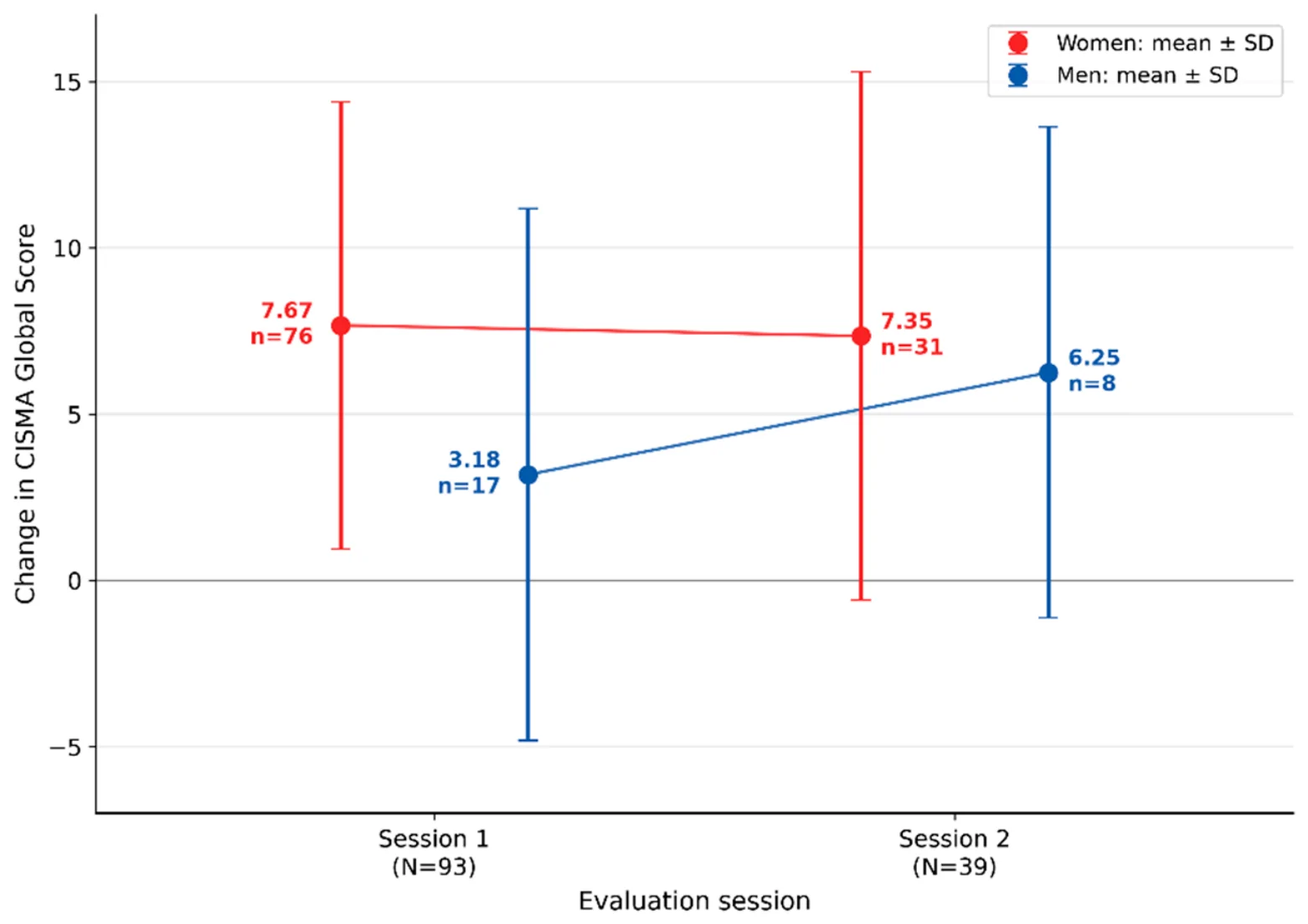
Changes in CISMA score according to participants’ sex. The figure presents the differences in CISMA score changes observed between male and female participants across the study sessions. Differences were evaluated by independent *t*-tests.

Although the global linear mixed-effects model indicated no significant main effect for sex (F(1, 82.13) = 2.84, *p* = 0.096) and no significant interaction terms, exploratory independent-samples *t*-tests were conducted on the change scores (Δ CISMA) to characterize specific, short-term variations. These sex-based analyses explore potential differences in the magnitude of pre–post score variations between male and female participants across the evaluation framework (Figure 5).

During the first session, female participants (n = 76) demonstrated a larger average descriptive increase in global scores, with a mean shift of 7.67 points (±6.72), whereas male participants (n = 17) exhibited an average variation of 3.18 points (±8.00). An independent-samples *t*-test detailed that this difference in the magnitude of change was statistically significant (t(91) = −2.41, *p* = 0.018, Cohen’s d = −0.65; 95% CI [−1.19, −0.11]), indicating a more pronounced descriptive upward variation among women immediately following the initial sound exposure.

In the second session, evaluated within the longitudinal sub-cohort, female participants (n = 31) displayed a descriptively higher mean score shift of 7.35 points (±7.94), while male participants (n = 8) showed a mean adjustment of 6.25 points (±7.38). However, the independent-samples *t*-test revealed that this mathematical divergence did not reach statistical significance (t(37) = −0.36, *p* = 0.724), and Cohen’s d reflected a negligible effect size (d = −0.14; 95% CI [−0.92, 0.64]).

Overall, within the limitations of this non-randomized, uncontrolled longitudinal study, the data suggested that while a steeper initial variation was observed among female participants, this sex-based divergence did not maintain statistical consistency across longitudinal exposure. Both groups ultimately experienced descriptive upward shifts in their well-being metrics. These findings should be interpreted with caution because of the small and unbalanced subgroup sizes, particularly among male participants in the longitudinal cohort.

### 3.5. Changes in the Pain Dimension of the CISMA Test in the Two Different Sessions

**Figure 6:**
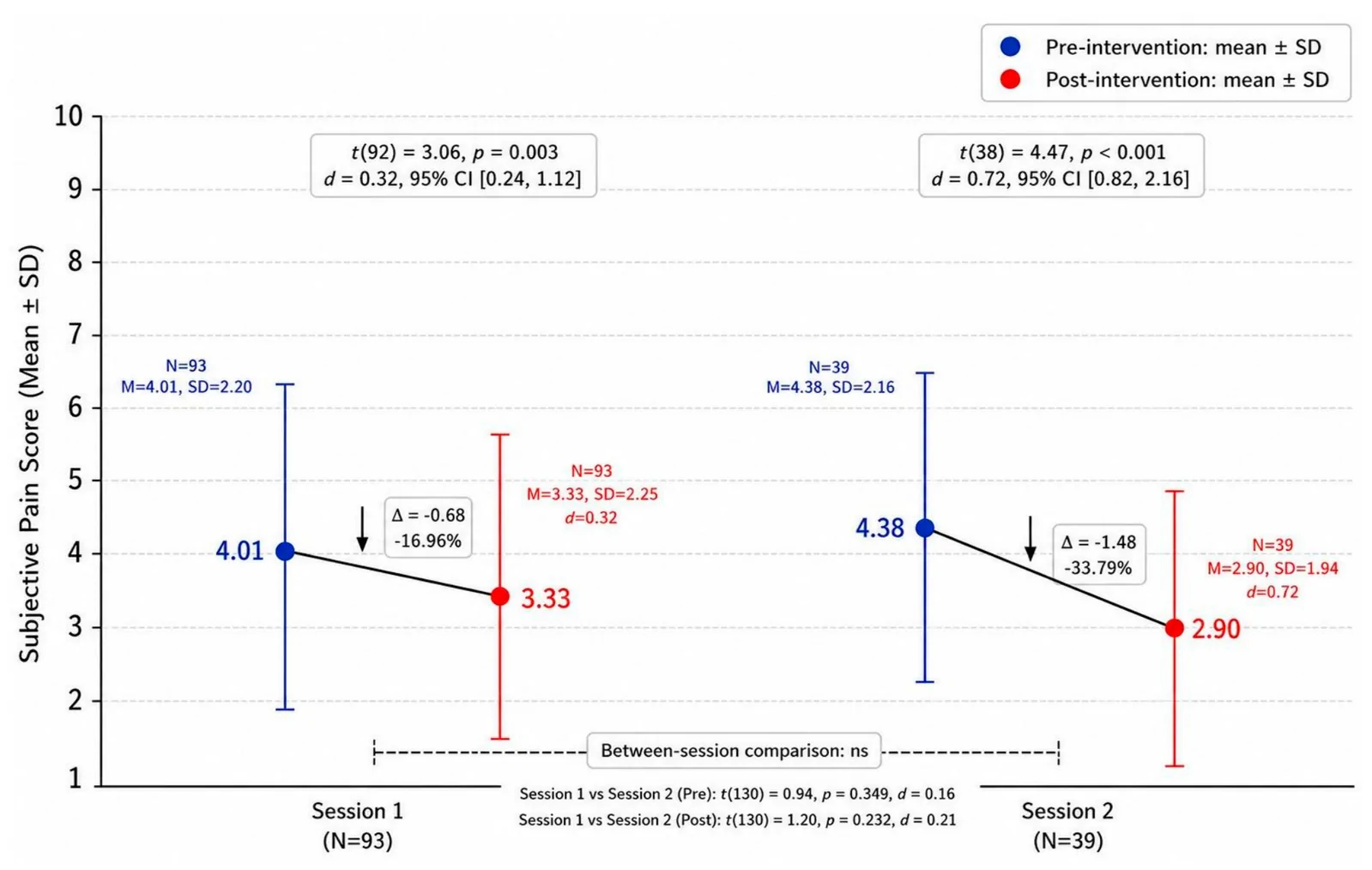
Changes in the pain dimension of the CISMA test across the two study sessions. The figure depicts the variation in pain-related CISMA scores obtained before and after each session. Differences were evaluated by paired *t* tests.

Complementing the primary insights derived from the global multi-factorial framework, specific exploratory analyses were performed on individual dimensions of the questionnaire. These dimension-specific scores represent individual CISMA components and should not be interpreted as the global CISMA score. Each dimension was analysed according to its own scoring procedure and range. Particular focus was placed on the descriptive dynamics of pain metrics (Δ Pain) to examine short-term variations across the evaluation periods, indicating a reduction in reported pain levels following the sessions in both encounters (Figure 6).

During the first session, the mean pre-exposure pain score stood at 4.01 points (±2.2) [on a scale of 1–10], which decreased to 3.33 points (±2.25) immediately after the intervention. This reduction of 0.68 points represented a 16.96% decrease in subjective pain perception. A paired-samples *t*-test detailed that this pre–post mathematical variation was statistically significant (t(92) = 3.06, *p* = 0.003), displaying a small-to-moderate standardized mean difference (Cohen’s d = 0.32, 95% CI of the difference [0.24, 1.12]), reflecting a substantial downward shift in subjective pain scores.

In the second session, pain perception also decreased significantly after the intervention. The mean pre-intervention score was 4.38 points (±2.16), while the post-intervention score dropped to 2.90 points (±1.94). This reduction of 1.48 points corresponded to a 33.79% decrease in perceived pain, representing a more pronounced descriptive variation compared to the initial session. The paired-samples *t*-test indicated the statistical significance of this divergence (t(38) = 4.47, *p* < 0.001), demonstrating a substantial standardized effect size (Cohen’s d = 0.72, 95% CI of the difference [0.82, 2.16]).

Overall, while the lack of an active control group prevents causal claims regarding clinical efficacy, these findings identify a consistent descriptive trend toward the short-term attenuation of subjective pain scores immediately following both interventions.

## 4. Discussion

This study explored the effects of music therapy (specifically, the modality employing gong sound therapy) on the well-being of adults with high sensory processing sensitivity (SPS), comparing the responses of highly sensitive persons (HSPs) and non-HSPs across two intervention sessions.

The findings show a mathematical shift in the post-intervention scores of the tracked sample regarding emotional regulation, anxiety reduction, and pain perception management. Overall, the results describe an upward variation in self-reported psychological and perceived physical well-being scores within the CISMA framework in highly sensitive and non-highly sensitive participants within this exploratory cohort [24,25]. However, due to the uncontrolled nature of this framework, these variations cannot be interpreted as clinical efficacy or a specific therapeutic observation attributable to the intervention itself.

One of the most relevant findings of this study was the statistically significant improvement observed in CISMA scores after each intervention session. Participants demonstrated substantial increases in post-intervention well-being scores during both the first and second sessions, suggesting an immediate positive association between gong sound sessions and perceived emotional and physical well-being. These findings align with the previous literature indicating that music therapy and sound-based interventions can facilitate emotional regulation, reduce stress, and improve overall quality of life [26,27]. The consistency of the results across both sessions reflects a reproducible trend over time, although the specific influence of the gong frequencies cannot be completely isolated from generic relaxation mechanisms within this single-group framework.

Importantly, no statistically significant differences were observed when comparing pre-intervention scores between the first and second sessions, nor between post-intervention scores across sessions. These data suggest that participants presented relatively stable baseline reports during the evaluation period and that the post-session scores followed a consistent pattern across both time points. While this consistency suggests a reproducible immediate response within the sample, the lack of a control group prevents us from concluding whether this stability reflects an intervention-specific pattern or a routine accommodation to the testing environment. Consequently, rather than validating the overall design against external variables, these observations primarily highlight a shared trend in short-term post-session reports across exposures within this exploratory cohort [28,29,30].

Several psychological and theoretical biophysical mechanisms have been proposed to explain responses to sound-based interventions. However, the present study did not include physiological measurements and therefore cannot determine whether these mechanisms contributed to the observed changes. While these frameworks offer a plausible explanation for the positive trends in emotional well-being and pain reduction reported by participants, further comparative research is needed to determine whether these specific vibrational mechanisms provide therapeutic value beyond the natural relaxation induced by the setting itself [31,32].

The findings related to HSPs are particularly relevant considering the growing scientific interest in Sensory Processing Sensitivity (SPS) as a biologically based personality trait. SPS has been associated with deeper cognitive processing, heightened emotional responsiveness, and increased sensitivity to environmental stimuli [28,30]. Due to these characteristics, HSPs are often more vulnerable to overstimulation, emotional exhaustion, and stress-related symptoms [33,34]. Consequently, exploratory approaches aimed at fostering emotional balance and relaxation could offer meaningful insights into supportive strategies for this population, although further research with rigorous comparison groups remains necessary to confirm their specific benefits.

In the present study, both HSP and non-HSP participants showed significant improvements after the intervention. During the first session, no statistically significant differences were found between groups, suggesting a comparable pattern of post-session responses across participants, irrespective of their baseline sensitivity levels. This finding may indicate that the relaxing and regulatory experience associated with these sessions is perceived similarly across different sensory processing profiles within this sample. However, during the second session, a trend toward greater improvement was observed in the non-HSP group, although this difference did not reach statistical significance [25].

Several interpretations may explain this result. First, it is possible that HSP participants, due to their heightened sensory responsiveness, reached a therapeutic ceiling effect earlier than non-HSP individuals. Since HSPs process sensory information more deeply, they may have experienced intense emotional and subjective sensory responses during the first session, leaving less room for measurable improvement in subsequent interventions [12,35]. Conversely, non-HSP participants may have required repeated exposure to fully engage with the practice, which could explain the trend toward more pronounced score variations during the second session.

Another possible explanation relates to the complexity of sensory processing in HSPs. Although highly sensitive individuals often report positive responses to supportive and calming environments, they may also be more susceptible to sensory overstimulation, even in therapeutic contexts [35]. Gong sound therapy involves intense vibrational and auditory stimulation, which could evoke both relaxing and activating effects simultaneously [36,37]. Therefore, some HSP participants may have undergone deeper emotional processing that was not fully captured through the CISMA questionnaire alone.

The sex-related findings also deserve consideration. Women demonstrated significantly greater increases in well-being reports than men during the first intervention session, while similar trends persisted during the second session without reaching statistical significance. These results align with previous research indicating that women often report stronger emotional engagement and responsiveness within music-based frameworks [38]. Women may also display greater emotional expressiveness and openness to introspective therapeutic experiences, potentially facilitating deeper immersion in sound therapy sessions. However, the interpretation of these findings should be approached cautiously. The lack of statistical significance during the second session suggests that gender differences may not represent a stable or universal effect. Additionally, the unequal distribution of men and women within the sample may have influenced the statistical power of the analyses [39].

One of the most notable observations of the present study concerns the significant reduction in pain perception observed after both intervention sessions. Participants reported consistently lower subjective pain scores following the gong sound therapy, with reductions reaching 16.96% in the first session and 33.79% in the second session. In both cases, these reductions were highly statistically significant, highlighting a favorable trend in alleviating subjective pain perception within this sample. These findings are particularly important given the growing interest in non-pharmacological and complementary approaches for pain management, although further controlled trials are necessary to verify whether these reductions are specifically driven by the acoustic intervention or secondary to natural muscle relaxation [40].

The relationship between music therapy and pain reduction has been documented in multiple healthcare contexts. Previous studies suggest that auditory stimulation may reduce pain perception through attentional distraction, emotional modulation, and relaxation responses [41,42]. However, the physiological mechanisms underlying these effects were not assessed in the present study. In the case of gong therapy, low-frequency vibrations may additionally induce somatic resonance experiences associated with variations in bodily awareness and muscular tension. The deep relaxation reported during these sessions may therefore share a link with the decreased subjective pain intensity observed within the sample [17].

From a clinical perspective, these findings have significant implications. Contemporary healthcare systems increasingly recognize the need for holistic and patient-centered approaches that address emotional and psychological dimensions alongside physical symptoms [22,43]. Complementary therapies such as music therapy and sound healing may offer cost-effective, non-invasive, and accessible interventions associated with enhancements in patient well-being while reducing dependence on pharmacological treatments [44]. The use of gong sound therapy can be especially relevant in nursing contexts characterized by high emotional demands, stress, and a risk of burnout. Both patients and healthcare professionals, particularly those with high sensory sensitivity, could find value in frameworks that foster relaxation, and emotional regulation, although this requires confirmation in appropriately designed studies. In this sense, while limited by its exploratory nature, the present study provides preliminary insights that encourage further discussion regarding the integration of complementary approaches into holistic care models [30].

To provide a solid scientific foundation for the proposed mechanisms of action, the recent clinical literature (including high-quality randomized controlled trials (RCTs), systematic reviews, and meta-analyses in the fields of music therapy and sound-based interventions) consistently supports the plausibility of these hypotheses [45]. Various controlled clinical studies have demonstrated that structured music listening and sound frequencies act as neurophysiological modulators [32].

The study also highlights the importance of environmental conditions within the context of the observed outcomes. The sessions were conducted in quiet and welcoming spaces, specifically adapted to facilitate relaxation and sensory well-being. These environmental considerations can be particularly important for highly sensitive individuals, who are more sensitive to noise, lighting, interpersonal tension, and environmental overstimulation.

### Limitations of the Study

Several limitations must be acknowledged when interpreting these findings. The primary constraint is the lack of a true passive or active control group, which prevents us from definitively decoupling the specific therapeutic effects of the gong’s vibrational frequencies from non-specific factors, such as placebo responses, participant expectations, time-dependent relaxation, or practice effects from repeated administrations of the CISMA questionnaire. Furthermore, because this study is strictly non-randomised, selection bias might have played an important role in the outcomes, as participants volunteered out of prior interest, which restricts the generalizability of the observed data. While these preliminary results provide encouraging trends of emotional and pain modulation, randomized controlled trials with a dedicated control group are strictly required to establish causality.

Additionally, the substantial attrition observed across the evaluation framework—manifested as a 28.5% within-session data exclusion in the first session (N = 130 to N = 93) due to incomplete assessments, followed by a 58% longitudinal dropout from Session 1 to Session 2 (n = 93 to n = 39)—introduces a prominent risk of attrition bias. Although our post-hoc power analysis confirmed that the final samples were fully adequate to achieve maximum statistical power (1 − β ≈ 1.0) for the primary pre–post longitudinal contrasts, the elevated dropout rate significantly reduced the sizes of specific sub-cohorts (particularly the non-HSP and male cohorts). Consequently, as detailed by the post-hoc metrics (1 − β ≈ 0.70 for cross-sectional contrasts), the statistical power for secondary subgroup and interaction analyses was moderately compromised, which limits the generalizability of these specific repeated-exposure findings. This longitudinal loss was primarily driven by scheduling conflicts and participant unavailability for the designated second session date, representing a logistical limitation rather than a systematic dropout linked to the intervention’s efficacy or adverse effects. Nevertheless, because the specific representativeness of the remaining 39 completers cannot be statistically guaranteed, all longitudinal metrics from the second session must be interpreted with strict caution.

Another logistical limitation arose from evaluating the sub-cohorts in different physical locations due to institutional constraints, as the HSP association headquarters were exclusively restricted to registered members, requiring the non-HSP group to be assessed in a multipurpose training room. This geographical split introduces a complete confounding variable between group membership and environmental setting, meaning that any statistical analysis cannot mathematically separate the psychological traits of the participants from the context of the venue. Although the physical micro-environment (including floor insulation mats, dimmed lighting, uniform temperature control, and strict ambient noise mitigation) was completely standardized across both spaces, this control cannot account for the social, institutional, and emotional meaning of the space—such as familiarity, expectancy, and sense of belonging—which are particularly salient for highly sensitive individuals. Consequently, the statistical uniformities in baselines and the parallel improvement trajectories observed between cohorts cannot rule out this confounding effect. Therefore, the comparison between the HSP and non-HSP groups must be interpreted as strictly descriptive, as the setting effect and the group effect remain inseparable. Future independent replications should utilize single-site experimental environments to completely isolate individual psychological traits from macroscopic environmental variances.

## 5. Conclusions

In conclusion, this study suggests that music therapy, specifically utilizing gong-based sound experiences, is associated with positive self-reported changes for both highly sensitive people (HSPs) and non-HSPs. In both sessions, significant variations were observed in post-intervention scores compared to pre-intervention scores. These findings suggest that gong-based music therapy represents a promising complementary framework applicable to individuals with varying levels of sensitivity, potentially contributing to immediate shifts in emotional and psychological well-being. However, due to the exploratory nature of this single-group pre–post design, these insights warrant further validation through controlled experimental methodologies to fully establish their specific therapeutic efficacy.

The study results also indicate that the intervention was associated with a significant reduction in subjective pain perception, with clearly observable decreases between pre- and post-intervention measurements across both sessions. This highlights the potential utility of sound-based music therapy as a complementary tool for subjective pain modulation, suggesting relevant implications for its implementation in clinical and wellness settings. Nevertheless, in the absence of an active control group, these preliminary observations of pain attenuation must be interpreted with strict caution and require formal confirmation through controlled experimental trials before any causal or clinical efficacy claims can be made.

Analysis of individual variables revealed differences in response to the intervention according to sex and level of high sensitivity, although not all reached statistical significance. In the first session, women showed significantly greater score variations than men, and this trend continued in the second session, although the differences were not statistically significant. Regarding sensitivity, both HSP and non-HSP participants demonstrated comparable and significant improvements during the initial session; however, during the second exposure, the non-HSP group displayed a trend toward more pronounced score variations that approached the threshold of statistical significance. These findings suggest that there are individual variations in the responses to this music therapy intervention, possibly related to personal characteristics such as gender and sensitivity. Nevertheless, further randomized trials with larger, balanced samples and extended follow-up periods are strictly required to validate these descriptive trends and support their integration into clinical practice.

## Figures and Tables

**Table 1:** Baseline demographic and clinical characteristics of the study sample (N = 93). Note: HSP = Sensory Processing Sensitivity/Highly Sensitive Person; SD = Standard Deviation. Baseline scores represent pre-intervention measurements during the first session. Both male and female cohorts participated in the study, displaying statistically homogenous distributions at baseline.

Characteristic	Total Sample (N = 93)	HSP Group (n = 62)	Non-HSP Group (n = 31)
Age (years)			
Mean ± SD	44.97 ± 11.21	44.20 ± 11.50	46.50 ± 10.60
Range	18–67	18–67	21–65
Sex, n (%)			
Female	76 (81.7%)	51 (82.3%)	25 (80.6%)
Male	17 (18.3%)	11 (17.7%)	6 (19.4%)
Baseline CISMA Scores			
Global Well-being (Mean ± SD)	22.90 ± 7.20	22.45 ± 7.42	23.80 ± 6.88

## Data Availability

The data presented in this study are available upon request to the corresponding author due to privacy reasons.
